# 

**Published:** 1892-11

**Authors:** 


### CONTENTS.

ORIGINAL COMMUNICATIONS—

The Present Demand for Better Medical Education in the South. By Luther B. Grandy, M. D., Atlanta, Ga. .... 513
Clinical Observations on Continued Fever Occurring in this Climate. By Everard H. Richardson, M. D., Atlanta, Ga. .... 523
Empyema. By P. O'Keef, M. D., Oconto, Wis. .... 530
Modern Electrical Methods as a Substitute for Surgery in Certain Pelvic Affections. By G. Betton Massey, M. D., Philadelphia, Pa. .... 535

SOCIETY REPORT—

Allegheny County Medical Society .... 537
American Association of Obstetricians and Gynecologists .... 540

CORRESPONDENCE—

From New York .... 543
Fatal Delay in a Case of Obstetrics .... 545

EDITORIAL—

Some Legislation That We Need .... 546

Medical Journals and Advertising .... 550

"Cœliotomy," not "Laparotomy" .... 551

SELECTIONS .... 552

MEDICAL ITEMS .... 57[illegible]

BOOK REVIEWS .... 575

INDEX TO ADVERTISEMENTS .... xii

ITEMS OF INTEREST .... x-xi

